# The impact of Regional co-payment and National reimbursement criteria on statins use in Italy: an interrupted time-series analysis

**DOI:** 10.1186/1472-6963-14-6

**Published:** 2014-01-06

**Authors:** Gianfranco Damiani, Bruno Federico, Angela Anselmi, Caterina Bianca Neve Aurora Bianchi, Giulia Silvestrini, Lanfranco Iodice, Pierluigi Navarra, Roberto Da Cas, Roberto Raschetti, Walter Ricciardi

**Affiliations:** 1 Department of Public Health, Catholic University of the Sacred Heart, Rome, Italy; 2 Department of Human Sciences, Society and Health, University of Cassino and Southern Lazio, Cassino, Italy; 3 Department of Pharmacology, Catholic University of the Sacred Heart, Rome, Italy; 4 National Institute of Health, Rome, Italy

**Keywords:** Interrupted time-series, Statin use, Pharmaceutical policy, Italy

## Abstract

**Background:**

Statins are among the most commonly prescribed drugs worldwide in the prevention of cardiovascular diseases and their effectiveness is largely acknowledged. The consumption of statins increased four-fold during the 2000–2010 decade in Italy and national and regional control policies were developed. Restrictions to reimbursement were fixed at the national level, whereas co-payment was introduced in some, but not all, regions. The aim of the present study is to assess the impact of such policies on the consumption of statins in Italy between 2001–2007 among outpatients.

**Methods:**

The statin use was measured in terms of defined daily doses per 1,000 inhabitants per day (DDD/1000 inh. day) from May 2001 to December 2007. The study was conducted in 17 out of 21 regions, nine of which had implemented a co-payment policy. Time trends in consumption before and after the introduction of co-payment policies and reimbursement criteria were examined using segmented regression analysis of interrupted time-series, adjusting for seasonal components.

**Results:**

The consumption of statins increased by 22.9 DDD/1000 inh. day in May 2001 to 54.7 DDD/1000 inh. day in December 2007. On average, there was a 1.7% increase in statin use each month before the national guideline changed while the increase was about 0.5% afterwards. The revision of the reimbursement criteria was associated with a significant decrease in level (coefficient = −2.80, 95% CI −3.70 to −1.90 p-value <0.001) and trend (coefficient = −0.33, 95% CI −0.37 to −0.29 p-value <0.001). The introduction of co-payment was associated with a significant change in trend of consumption so that the overall use of the drug increased by 0.04 (95% CI 0.02 to 0.07, p-value < 0.001) DDD/1000 inh. day per month in the post-intervention period, but there was no evidence of a change in level of consumption (p-value = 0.163).

**Conclusions:**

Consumption of statins in Italy increased almost three-fold during the study period. The restriction to reimbursement Interventions was associated with an immediate drop and a decrease in trend of statin use, while the regional copayment was associated with a small increase in trend of statin use.

## Background

In recent years, many policies have been implemented across the European Union to enhance the appropriate use of drugs and to contain pharmaceutical cost [1,2].

The 3-hydroxy-3-methylglutaryl coenzyme A (HMGCoA) reductase inhibitors (statins) were first used in patients with myocardial infarction (MI) and hypercholesterolemia. Several clinical trials, cohort studies and reviews largely acknowledged statins effectiveness: their beneficial effects were evident as lipid-lowering agents in patients with ischemic heart disease (IHD), other atherosclerotic cardiovascular diseases (CVD), diabetes, and even in the treatment of asymptomatic individuals [3-12]. These clinical trials had major implications for cholesterol management that resulted in an increase in the number of patients for whom statins may be considered clinically appropriate [13], such raising the expenditure, at least before the patent expiration of some molecules.

Thus, a wide range of measures to contain the cost of statins was developed in many Countries (e.g. Norway), including lists of preferred generic substitutions and/or medicines for which the patients are reimbursed (Austria and Finland), and co-payment policies (USA, Canada). The effect of these policies was assessed in recent studies with regards to drug expenditure, adherence therapy, or health outcomes [14-19].

Even though Italy has one of the lowest rates of use in Europe [20], the consumption of statins has constantly increased during the last decade, almost quadrupling its level from 14.67 DDD/1000 inhabitants per day (DDD/1000 inh. day) in 2000 to 51.70 DDD/1000 inh. day in 2010 [21,22]. Compared to 2010, in 2011 (the latest available data for a whole year) statin consumption still increased (+7.7%) while the expenditure decreased (−13.1%) Despite this fact, statins rank first in expenditure as a cardiovascular drug subgroup [23].

Most of the costs are charged to the Italian National Health Service (Servizio Sanitario Nazionale, SSN) which provides universal coverage.

In order to contain the cost of drugs, a co-payment policy was first introduced in Italy in 1978, then abolished in January 2001 by the national government, and subsequently it was reintroduced at the local level by some regions, following the decentralization trend [24] of the policy-making government.

The change in Italian Constitutional Chart in 2001, determined a devolution process that transferred legislative, administrative and considerable fiscal powers to regions. According to this, the central government was granted the exclusive power to set “essential levels of care” (Livelli Essenziali di Assistenza, LEAs), a package of benefits that are publicly funded and must be guaranteed to all citizens in all regions. Most essential drugs are included in LEAs. At the same time, regions have almost full control over the provision of services, regulation, and funding with the mandate to provide LEAs [25]. LEAs hold regions accountable to national standards.

Thus, since 2002, regions have made different choices regarding co-payment policies with subsequent significant cross-regional variation among patient charges for drugs [26]. In regions with a co-payment policy, patients are required to contribute to the cost of pharmaceuticals by a fixed amount per prescription, ranging from 1 to 5 euros, depending on the region [27]. On the other hand, the criteria for the reimbursement of statins were issued and periodically revised at the national level. They contained specific indications, inspired by clinical practice and guidelines, that determined whether the statins could be prescribed and reimbursed for specific diseases and conditions [28]. Their introduction had a dual purpose: cost containment and a more appropriate use of these drugs [29]. In November 2004, according to new scientific evidence, criteria for reimbursement for lipid lowering agents were revised by the new Italian Drug Agency (AIFA). The new criteria (Nota AIFA 13) restricted statin reimbursement to high risk users, based on a national risk profile for primary CVD prevention while they extended reimbursement for patients with diabetes in secondary CVD prevention [30,31].

The aim of this study was to evaluate the impact of the national and regional cost containment measures on statin use in Italy during the period May 2001 – December 2007.

## Methods

### Data

Data on drug consumption in Italy between May 1, 2001 and December 31, 2007 were collected from the Federfarma database. This database includes all drug use paid by the National Health Service, excluding in-hospital as well as out-of-pocket drug use. Data were provided by the Italian National Institute of Health on an aggregate basis, with regions and time being the unit of analysis. Because no individual data was available, ethical approval of the study was not sought.

The dataset included the number of Defined Daily Doses per 1000 inhabitants per day (DDD/1000 inh. day) for all drugs in the C10AAxx category of the Anatomical Therapeutic Chemical (ATC) system [32]. Data from each Italian region were available on a monthly basis, and they were adjusted by age and sex according to the weights established by the Department of Programming of the Italian Ministry of Health.

The analysis considered 17 out of the 21 Italian regions. They were divided in two groups. The first one included regions which (re)introduced a co-payment: Bolzano, Campania, Liguria, Lombardia, Molise, Piemonte, Puglia, Sicilia and Veneto. The second one included those regions that did not reintroduce a co-payment: Basilicata, Emilia Romagna, Friuli Venezia Giulia, Marche, Toscana, Trento, Umbria, Valle d’Aosta. We excluded Abruzzo, Calabria, Lazio and Sardegna because these regions modified the co-payment mechanism several times during the study period.

### Statistical analysis

A descriptive analysis of the consumption of statins during the study period was first performed. Tables describing the consumption of statins for both regions with and without co-payment policies have been compiled. An average monthly variation index (*calculated as: ****[(End-period consumption/Start-period consumption)***^***1/n***^− 1]*100) was provided for both the periods pre- and post- prescribing guideline change. Graphs showing the statins use trends for both regions with and without co-payment policies were plotted. Subsequently, we analysed the series to identify the most appropriate model to describe the data until the time of intervention, and to make a forecast for the post-intervention period in order to asses if there was a significant impact of the policy change on the consumption of statins. We chose the most befitting model (between the ARIMA and the exponential smoothing models) after a residual analysis through the Ljung-Box test [33] and the check of the autocorrelation and partial autocorrelation functions of the residual. The mean absolute error (MAE) and the mean absolute percentage error (MAPE) between the observed and the predicted series were also calculated in order to identify the best forecasting technique for the data as the model that minimized these forecast indices.

The model of choice was the Holt-Winters Additive model, that was the best forecasting technique among the Exponential smoothing models (MAE = 1.516; MAPE = 4.727). This final model was the result of several iterations of the identification, estimation, and checking process, and it met the conventional criteria for the adequacy of the model. The autocorrelation and partial autocorrelation functions of the residuals showed a good fit. The residual plots showed small variations around the zero mean. None of these residuals had its magnitude larger than twice the standard deviation. Residual autocorrelations were not significantly different from zero as a set and had constant variance, thus confirming the adequacy of the model (Ljung-Box statistic = 6.807; p-value = 0.963).

To determine the extent of the impact of policy changes on pharmaceutical utilisation, we used segmented linear regression for the interrupted time-series analysis [34]. This analysis provided estimates of an outcome (DDD/1000 inh. day) before and after defined change points (the introductions of co-payment and the revision of national prescription guidelines). To eliminate the seasonal component related to the consumption of the drug and assess the “true effect” of the above-mentioned measures, we performed a seasonal decomposition on the series before the estimation of the regression model. Segmented linear regression divides a time-series into pre- and post-intervention segments. In this case, we had two change points at different time periods, so our series was divided into three segments.

The model has two parameters of interest: the level and slope. Therefore the difference between the two segments can be quantified by testing the change in these two parameters. A change in level between the pre- and the post-intervention segments indicates a step-change, and a change in slope indicates a change in trend. The estimated base model is the following:

(1) $$\begin{array}{l} Y_{t}=\beta_{0}+\beta_{1}*\text{time}+\beta_{2}*\mathrm{copayment}+\beta_{3}\\ \;*\text{time}\;\text{after}\;\text{copayment}+\beta_{4}*\text{reimbursement}\\ \;+\beta_{5}*\text{time}\;\text{after}\;\text{reimbursement}+\sum_{i=1}^{16}\beta_{i}*D_{i}+e_{t} \end{array}$$

Here, Y_t_ is the mean number of DDD/1000 inh. day in month t; *time* is a continuous variable indicating time in months at time t from the start of the observation period; *co-payment* is an indicator for time t occurring before (intervention = 0) or after (intervention = 1) its introduction, which occurred at different instants of time for the considered Italian regions; *time after co-payment* is a continuous variable counting the number of months after the intervention at time t, coded 0 before the introduction and 1 (time - time of intervention) after. *Reimbursement* and *time after reimbursement* are two variables built in the same mode of the previous ones but refer to the revision of national reimbursement criteria in November, 2004; e_t_ is the error term at time t and represents the random variability not explained by the model. In this model, β_0_ estimates the baseline level of the outcome (expressed in DDD/1000 inh. day) per month, at time zero; β_1_ estimates the pre-intervention slope (i.e. the baseline trend); β_2_ estimates the level change immediately after the introduction of co-payment, that is from the end of the preceding segment; β_3_ estimates the change in trend relative to the first intervention compared with the pre-existing monthly trend; β_4_ and β_5_ are respectively the estimate of the level and trend change relative to the revision of AIFA Notes. D represents 17–1 Dummy variables relative to the considered regions. We coded the regional Dummies by the “deviation from means coding” method [35]. This coding expresses effect as the deviation of the “group mean” from the “overall mean”.

We tested whether each of these parameters is significantly different from zero from a two-sided *t* test to determine whether a discontinuity at each join point existed.

For statistical analysis we used the software SPSS v.13.0 for Windows environment.

## Results

**Figure 1:**
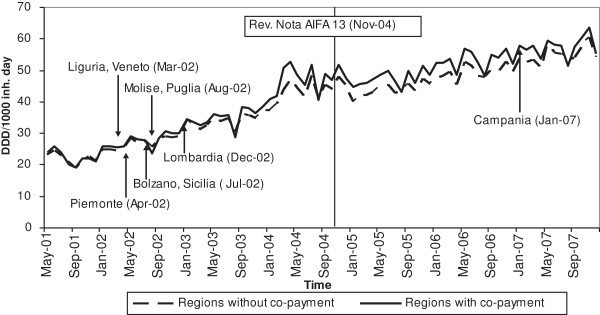
**Monthly statins consumption (DDD/1000 inh. day) in the two groups of regions.** The arrows show the month and the year in which the indicated regions have applied the co-payment measures.

Figure 1 shows the time series of the mean number of statins use (DDD/1000 inh. day) per month, interrupted by the two policy changes: the introduction of co-payment in different dates for each regions, indicated by the arrows in the graph, and the revision of AIFA Notes in November 2004, indicated by the vertical line. The visual inspection of the series suggests that the use of statins increased during the study period for both groups of regions.

As shown in Table 1, the consumption of statins increased by 22.9 DDD/1000 inh. day in May 2001 to 54.7 DDD/1000 inh. day in December 2007. On average, there was a 1.7% increase in statin use each month before the national guideline changed while the increase was about 0.5% afterwards.

**Table 1:** Statins consumption per 1000 inhabitants per month in the considered Italian regions

	**Regions**	**May-01**	**Nov-04**	**Dec-07**	Δ% (04–01) *	Δ% (07–04) *
Regions with the co-payment	P.A. Bolzano	23.83	40.25	42.99	1.26	0.17
	Campania	26.23	54.00	61.32	1.73	0.33
	Liguria	13.93	47.27	52.91	2.95	0.29
	Lombardia	27.29	48.81	55.83	1.39	0.35
	Molise	17.53	39.79	45.40	1.97	0.34
	Piemonte	23.90	39.62	47.74	1.21	0.48
	Puglia	21.61	47.06	63.55	1.87	0.77
	Sicilia	25.89	55.91	66.84	1.85	0.46
	Veneto	26.61	48.06	59.69	1.42	0.56
Total Regions with co-payment :		22.98	46.75	55.14	1.74	0.42
Regions without co-payment	Basilicata	20.93	50.00	58.43	2.10	0.40
	Emilia-Romagna	27.01	50.10	61.66	1.48	0.53
	Friuli Venezia Giulia	26.57	47.72	57.11	1.40	0.46
	Marche	21.38	45.47	62.00	1.81	0.80
	Toscana	21.60	38.21	49.24	1.37	0.65
	P.A. Trento	24.80	41.27	53.58	1.22	0.67
	Umbria	19.16	39.83	50.50	1.76	0.61
	Valle d’Aosta	21.46	40.73	41.86	1.54	0.07
Total Regions without co-payment:		22.86	44.17	54.30	1.58	0.52
Total:		22.93	45.54	54.74	1.67	0.47

Comparison May, 2001- November, 2004 and November, 2004- December, 2007.

*Average monthly variation index.

**Figure 2:**
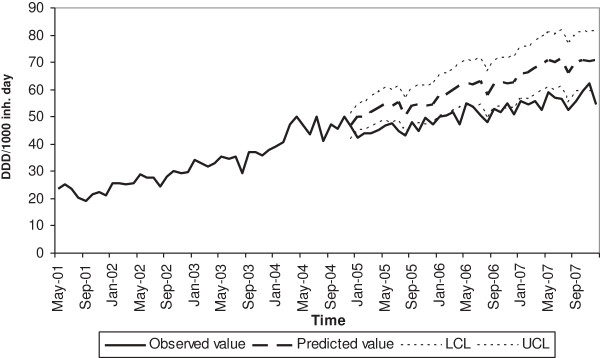
**Monthly statins consumption (DDD/1000 inh. day) in the two groups of regions.** Observed vs predicted. The figure shows observed vs predicted series after the change of the reimbursement criteria in November 2004. Lower and upper confidence level are set at 95%.

As shown in Figure 2, the revision of AIFA Note 13 was associated with a large change in the use of statins because the observed series is outside the 95% confidence interval for the short-term forecast. The short-term forecast beyond the observed series (from May 2001 to November 2004) of statin consumption in all the seventeen regions adequately captured the pattern in the data until the policy change (revision of AIFA Notes) and showed an increasing tendency of drug use with a mean 9.7 DDD/1000 inh. day each month over the observed series during the forecast interval (post intervention period).

This result was confirmed by the estimates of the regression model that explained the effect of policy change. Table 2 contains the parameter estimates from the linear segmented regression model.

**Table 2:** Model parameter estimates, standard errors and p-values of statins consumption

**Model**	**Coefficients**	**p-value**	**95% Conf. interval**	
	**B**		**Lower**	**Upper**
(Constant)	18.06	< 0.001	17.43	18.69
time	0.68	< 0.001	0.65	0.71
Co-payment	−0.78	0.163	−1.88	0.32
Time after co-payment	0.04	< 0.001	0.02	0.07
Reimbursement criteria change	−2.80	< 0.001	−3.70	−1.90
Time after reimbursement criteria change	−0.33	< 0.001	−0.37	−0.29

The introduction of co-payment was associated with a significant change in slope so that the overall use of the drug increased by 0.04 (95% CI 0.02 to 0.07, p-value < 0.001) DDD/1000 inh. day per month in the post-intervention period, but there was no evidence of a change in level (p-value = 0.163).

Prescription guidelines showed a large impact on the use of statins. A temporal association between the revision of the AIFA Note in November 2004 and a remarkable reduction in the upward trend of statin use in the same period were observed. This intervention was associated with a significant decrease in level (coefficient = −2.80, 95% CI −3.70 to −1.90 p-value <0.001) and slope (coefficient = −0.33, 95% CI −0.37 to −0.29 p-value <0.001).

## Discussion

This study showed that the change in the national reimbursement criteria influenced the utilization of statins in Italy, determining a statistically significant reduction in the upward trend of prescriptions. On the other hand, the effect of the local co-payment on statin use was negligible. Overall, statin consumption increased from 2001 to 2007, according to previous research showing a similar trend in most European countries [36].

Reimbursement criteria revision that occurred in Italy in 2004 was issued with the aim to improve the appropriateness of drug prescriptions, following updated scientific evidence related to cardiovascular disease risk reduction. Our findings are consistent with Trifirò et al. [37] that reported a significant decrease in the utilization of statin after the Nota 13 change in a general practice in southern Italy. An increased consumption was found only for diabetic patients, which was probably related to the extended criteria of reimbursement for secondary prevention. On the contrary, a study conducted in Austria by Winkelmayer et al. [15], showed that national change in statin prescribing policies from secondary to primary prevention among diabetic patients did not affect the overall trend of statin use. Northern Europe brings instead some examples of how policies that limit the reimbursement of drugs can be a powerful tool for changing the use of medication. A Norwegian study, conducted in 2007 [14], showed an increase in the consumption of statins and, simultaneously, a decrease of the expenditure after initiating an intervention that forced physicians to prescribe simvastatin or an alternative and to carefully explain the need to use a different statin. Such a policy caused an increase in prescriptions of the less costly statins and an increase in consumption by first-time users while physicians continued to preserve the adherence to the therapy. Similar results came from Finland [16] that reduced costs by instituting policies promoting use of less expensive statins and restricting reimbursement of the more expensive atorvastatin, whose patent expired only in late 2011 [38].

Authors from Harvard and Canada [39] recently applied the interrupted time series study design to assess the impact of the prior authorization (a prescribing-limitation policy that allows the prescription of a non-preferred medication only after the approval from Medicaid) with contrasting results for the two groups regarding the consumption of statins. The policy was associated with a lower use of statins in Michigan, but the effect was negligible in Indiana where there had been little use of non-preferred medications before the policy was implemented.

In our study, the impact of the co-payment on the consumption trend of statins increased statin use in a statistically significant way, but its real effect was very small, as shown in Table 2. Some authors stressed the fact that the main purpose of cost sharing is to introduce incentives so that patients would be able to compare cost to expected outcomes [40]. In Italy, however, the cost shared by the patient is fixed and not proportional as in other countries such as USA or Canada [19]; therefore, this measure becomes irrelevant to a price-oriented behaviour by the consumer. Consequently, it becomes a mere cost-containment tool in the short run [29]. This could conceal some implications for the safety of the population, possibly modifying the adherence to treatment [41-43].

Indeed, Gibson [17] found that as statin cost-sharing levels increase, adherence to statin treatment falls. This could lead to health risks, increasing access to health services and eventually to an increase in expenditure [1,44,45].

### Limitations and strengths

Unfortunately, aggregated data do not allow us to collect information about clinical outcomes. For this reason, we cannot assess the appropriateness in the prescription and the adherence to the therapy. As a second limitation, we did not consider the generic market, the marketing strategies such as incentives offered to general practitioners, or the regional policies (e.g., limit on the number of prescriptions for recipe) that may have had an impact on the consumption of statins. A third limitation resides on the observational nature of this study, which does not allow establishing a causal relationship between the intervention and the change in the consumption trend of statins.

However, we would like to highlight the strengths of this study. We could rely on a large dataset covering the entire national territory for a 7-year period. The data are weighted by gender and age, allowing us to overcome differences in the demographic structure among regions. Furthermore, the interrupted time series regression analysis allowed us to obtain more valid estimates of the impact of co-payment and national reimbursement criteria on the utilization of statins compared to a pre-post design [1,34,46].

## Conclusions

Consumption of statins in Italy increased almost three-fold during the study period. Interventions at the central level (modification of reimbursement criteria) had an impact in mitigating the growth, showing a temporal association with an immediate drop and a decrease in trend of statin use, while the regional policies (copayment) were associated with a small increase in trend of statin use. In a heavily regulated market, efforts for decentralisation should be aimed at gaining better control over the consumption of statins at the regional level.

## Competing interests

The authors declare that there was no financial relationships with any organizations that might have an interest in the submitted work; no other relationships or activities that could appear to have influenced the submitted work.

## Authors’ contributions

GD & BF conceived of the study and participated in its design, PN & WR coordinated the study and revised it, AA participated in its design and performed the statistical analysis, LI carried out literature search and drafted the manuscript, CBNAB & GS helped to draft the manuscript and contributed to the interpretation of data, RDC & RR collected data and revised the draft. All authors read and approved the final manuscript.

## Pre-publication history

The pre-publication history for this paper can be accessed here:

http://www.biomedcentral.com/1472-6963/14/6/prepub

## References

[B1] Austvoll-Dahlgren A Aaserud M Vist G Ramsay C Oxman AD Sturm H Kösters JP Vernby A Pharmaceutical policies: effects of cap and co-payment on rational drug use Cochrane Database Syst Rev 2008 14 1 CD007017 18254125 10.1002/14651858.CD007017

[B2] Ess SM Schneeweiss S Szucs TD European healthcare policies for controlling drug expenditure. (Review) Pharmacoeconomics 2003 14 2 89 103 10.2165/00019053-200321020-00002 12515571

[B3] Gotto AM Grundy SM Lowering LDL cholesterol: questions from recent meta-analyses and subset analyses of clinical trial data issues from the Interdisciplinary Council on Reducing the Risk for Coronary Heart Disease, 9th Council Meeting Circulation 1999 14 E1 E7 10051310 10.1161/01.cir.99.8.e1

[B4] Hebert PR Gaziano JM Chan KS Hennekens CH Cholesterol lowering with statin drugs, risk of stroke, and total mortality. An overview of randomized trials JAMA 1997 14 313 321 10.1001/jama.1997.03550040069040 9228438

[B5] Sever PS Dahlof B Poulter NR Wedel H Beevers G Caulfield M Collins R Kjeldsen SE Kristinsson A McInnes GT Mehlsen J Nieminen M O’Brien E Ostergren J ASCOT investigators Prevention of coronary and stroke events with atorvastatin in hypertensive patients who have average or lower-than-average cholesterol concentrations, in the Anglo-Scandinavian Cardiac Outcomes Trial-Lipid Lowering Arm (ASCOT-LLA): a multicentre randomised controlled trial Lancet 2003 14 1149 1158 10.1016/S0140-6736(03)12948-0 12686036

[B6] Eussen SR van der Elst ME Klungel OH Rompelberg CJ Garssen J Oosterveld MH de Boer A de Gier JJ Bouvy ML A pharmaceutical care program to improve adherence to statin therapy: a randomized controlled trial Ann Pharmacother 2010 14 12 1905 1913 10.1345/aph.1P281 21119098

[B7] Lingsma HF Steyerberg EW op Reimer WJ S van Domburg R Dippel DW Netherlands Stroke Survey Investigators Statin treatment after a recent TIA or stroke: is effectiveness shown in randomized clinical trials also observed in everyday clinical practice? Acta Neurol Scand 2010 14 1 15 20 20047571 10.1111/j.1600-0404.2009.01247.x

[B8] Pedersen TR Kjekshus J Berg K Haghfelt T Faergeman O Faergeman G Pyörälä K Miettinen T Wilhelmsen L Olsson AG Wedel H Scandinavian Simvastatin Survival Study Group Randomised trial of cholesterol lowering in 4444 patients with coronary heart disease: the Scandinavian Simvastatin Survival Study (4S) Lancet 1994 14 8934 1383 13897968073

[B9] Colhoun HM Betteridge DJ Durrington PN Hitman GA Neil HA Livingstone SJ Thomason MJ Mackness MI Charlton-Menys V Fuller JH CARDS investigators Primary prevention of cardiovascular disease with atorvastatin in type 2 diabetes in the Collaborative Atorvastatin Diabetes Study (CARDS): multicentre randomised placebo-controlled trial Lancet 2004 14 9435 685 696 10.1016/S0140-6736(04)16895-5 15325833

[B10] Penning-van Beest FJ Termorshuizen F Goettsch WG Klungel OH Kastelein JJ Herings RM Adherence to evidence-based statin guidelines reduces the risk of hospitalizations for acute myocardial infarction by 40%: a cohort study Eur Heart J 2007 14 2 154 159 17158123 10.1093/eurheartj/ehl391

[B11] Ali R Alexander KP Statins for the primary prevention of cardiovascular events in older adults: a review of the evidence Am J Geriatr Pharmacother 2007 14 1 52 63 10.1016/j.amjopharm.2007.03.008 17608248

[B12] Ong HT The statin studies: from targeting hypercholesterolaemia to targeting the high-risk patient QJM 2005 14 8 599 614 10.1093/qjmed/hci093 16006501

[B13] Puig-Junoy J What is required to evaluate the impact of pharmaceutical reference pricing? Appl Health Econ Health Policy 2005 14 2 87 98 10.2165/00148365-200504020-00003 16162028

[B14] Sakshaug S Furu K Karlstad Ø Rønning M Skurtveit S Switching statins in Norway after new reimbursement policy – a nationwide prescription study Br J Clin Pharmacol 2007 14 476 481 10.1111/j.1365-2125.2007.02907.x 17441934 PMC2048565

[B15] Winkelmayer WC Asslaber M Bucsics A Burkhardt T Schautzer A Wieninger P Pogantsch M Brookhart MA Pharmacoeconomics Advisory Council of the Austrian Sickness Funds Impact of reimbursement changes on statin use among patients with diabetes in Austria Wien Klin Wochenschr 2010 14 3–4 89 94 20213375 10.1007/s00508-009-1292-6

[B16] Martikainen JE Saastamoinen LK Korhonen MJ Enlund H Helin-Salmivaara A Impact of restricted reimbursement on the use of statins in Finland a register-based study Med Care 2010 14 9 761 766 10.1097/MLR.0b013e3181e41bcb 20706164

[B17] Gibson TB Mark TL Axelsen K Baser O Rublee DA McGuigan KA Impact of statin copayments on adherence and medical care utilization and expenditure Am J Manag Care 2006 14 SP11 SP19 17173486

[B18] Pilote L Beck C Richard H Eisenberg MJ The effects of cost-sharing on essential drug prescriptions, utilization of medical care and outcomes after acute myocardial infarction in elderly patients CMAJ 2002 14 3 246 252 12186169 PMC117469

[B19] Schneeweiss S Patrick AR Maclure M Dormuth CR Glynn RJ Adherence to statin therapy under drug cost sharing in patients with and without acute myocardial infarction: a population-based natural experiment Circulation 2007 14 16 2128 2135 10.1161/CIRCULATIONAHA.106.665992 17420348

[B20] Dickinson M Jacobzone S Pharmaceutical use and expenditure for cardiovascular disease and stroke: a study of 12 OECD countries 2003 OECD Health Working Papers, No. 1, OECD Publishing http://dx.doi.org/10.1787/237273475872

[B21] Gruppo di lavoro OsMed L’uso dei farmaci in Italia. Rapporto nazionale anno 2000 2001 Roma: Il Pensiero Scientifico Editore [OsMed Working Team. The drug utilisation in Italy, National Reports 2000]

[B22] Gruppo di lavoro OsMed L’uso dei farmaci in Italia. Rapporto nazionale anno 2010 2011 Roma: Il Pensiero Scientifico Editore [OsMed Working Team. The drug utilisation in Italy, National Reports 2011]

[B23] Gruppo di lavoro OsMed L’uso dei farmaci in Italia. Rapporto nazionale anno 2011 2012 Roma: Il Pensiero Scientifico Editore [OsMed Working Team. The drug utilisation in Italy, National Reports 2012]

[B24] Ghislandi S Krulichova I Garattini L Pharmaceutical policy in Italy: towards a structural change? Health Policy 2005 14 53 63 10.1016/j.healthpol.2004.02.012 15760698

[B25] Torbica A Fattore G The “Essential Levels of Care” in Italy: when being explicit serves the devolution of powers Eur J Health Econ 2005 14 Suppl 1 46 52 16258750 10.1007/s10198-005-0318-x PMC1388084

[B26] Mapelli V Lucioni C Spending on pharmaceuticals in Italy: macro constraints with local autonomy Value Health 2003 14 Suppl 1 S31 S45 12846924 10.1046/j.1524-4733.6.s1.4.x

[B27] Fiorio CV Siciliani L Co-payments and the demand for pharmaceuticals: evidence from Italy Econ Model 2010 14 835 841 10.1016/j.econmod.2009.07.019

[B28] Agenzia Nazionale del Farmaco (AIFA). Note AIFA http://www.agenziafarmaco.gov.it/it/content/note-aifa

[B29] Fattore G Jommi C The last decade of Italian pharmaceutical policy: instability or consolidation? Pharmacoeconomics 2008 14 1 5 15 10.2165/00019053-200826010-00002 18088155

[B30] Gruppo di lavoro OsMed Il Pensiero Scientifico L’uso dei farmaci in Italia. Rapporto nazionale anno 2004 2005 Roma: OsMed Working Team. The drug utilisation in Italy, National Reports 2005

[B31] Ferrario M Chiodini P Chambless LE Cesana G Vanuzzo D Panico S Sega R Pilotto L Palmieri L Giampaoli S CUORE Project Research Group Prediction of coronary events in a low incidence population. Assesing accurancy of CUORE Cohort Study prediction equation Int J Epidemiol 2005 14 2 413 421 10.1093/ije/dyh405 15659467

[B32] ATC/DDD Index 2013 http://www.whocc.no/atc_ddd_index/

[B33] Yaffee RA McGee M An introduction to time series analysis and forecasting: with applications of SAS® and SPSS® 2000 New York: Academic Press

[B34] Wagner AK Soumerai SB Zhang F Ross-Degnan D Segmented regression analysis of interrupted time series studies in medication use research J Clin Pharm Ther 2002 14 4 299 309 10.1046/j.1365-2710.2002.00430.x 12174032

[B35] Hosmer W Lemeshow S Applied Logistic Regression 2000 2 New York: Wiley

[B36] Walley T Folino-Gallo P Stephens P Van Ganse E Trends in prescribing and utilization of statins and other lipid lowering drugs across Europe 1997–2003 Br J Clin Pharmacol 2005 14 543 551 10.1111/j.1365-2125.2005.02478.x 16236045 PMC1884951

[B37] Trifirò G Alaqua M Corrao S Moretti S Tari DU Galdo M Caputi AP Arcoraci V UVEC Group Lipid-lowering drug use in Italian primary care: effects of reimbursement criteria revision Eur J Clin Pharmacol 2008 14 6 619 625 10.1007/s00228-007-0459-1 18213473

[B38] IMS Health, MIDAS, Dec 2011 http://www.imshealth.com/ims/Global/Content/Insights/IMS%20Institute%20for%20Healthcare%20Informatics/IHII_Medicines_in_U.S_Report_2011.pdf

[B39] Lu CY Law MR Soumerai SB Graves AJ LeCates RF Zhang F Ross-Degnan D Adams AS Impact of prior authorization on the use and costs of lipid-lowering medications among Michigan and Indiana dual enrollees in Medicaid and Medicare: results of a longitudinal, population-based study Clin Ther 2011 14 1 135 144 10.1016/j.clinthera.2011.01.012 21397779 PMC3980661

[B40] Smith DG The effects of copayments and generic substitution on the use and costs of prescription drugs Inquiry 1993 14 2 189 198 8314607

[B41] Poluzzi E Strahinja P Lanzoni M Vargiu A Silvani MC Motola D Gaddi A Vaccheri A Montanaro N Adherence to statin therapy and patients’ cardiovascular risk: a pharmacoepidemiological study in Italy Eur J Clin Pharmacol 2008 14 425 432 10.1007/s00228-007-0428-8 18176802

[B42] Lucioni C Mazzi S Cerra C Lottaroli S Dellagiovanna M Fratino P Uno studio di drug utilisation delle statine nella recente prassi terapeutica italiana Pharmacoeconomics 2006 14 1 3 17

[B43] Peterson AM McGhan WF Pharmacoeconomic impact of non-compliance with statins Pharmacoeconomics 2005 14 1 13 25 10.2165/00019053-200523010-00002 15693725

[B44] Goldman DP Joyce GF Zheng Y Prescription drug cost sharing: associations with medication and medical utilization and spending and health JAMA 2007 14 1 61 69 10.1001/jama.298.1.61 17609491 PMC6375697

[B45] Li X Guh D Lacaille D Esdaile J Anis AH The impact of cost sharing of prescription drug expenditures on health care utilization by the elderly: own- and cross-price elasticities Health Policy 2007 14 3 340 347 10.1016/j.healthpol.2006.11.002 17134787

[B46] Damiani G Federico B Silvestrini G Bianchi CB Anselmi A Iodice L Ronconi A Navarra P Da Cas R Raschetti R Ricciardi W Impact of regional copayment policy on selective serotonin reuptake inhibitor (SSRI) consumption and expenditure in Italy Eur J Clin Pharmacol 2013 14 4 957 963 10.1007/s00228-012-1422-3 23090700

